# Quantifying sputum production success during community-based screening for TB

**DOI:** 10.5588/ijtldopen.24.0319

**Published:** 2024-11-01

**Authors:** P.J Kitonsa, J. Sung, D. Isooba, S. Birabwa, I. Naluyima, J. Kakeeto, W. Kamya, A. Nalutaaya, P. Biché, D.W. Dowdy, A. Katamba, E.A. Kendall

**Affiliations:** ^1^ Uganda Tuberculosis Implementation Research Consortium, Walimu, Kampala, Uganda;; ^2^ Division of Infectious Diseases, Johns Hopkins University School of Medicine, Baltimore, MD, USA;; ^3^ Department of Epidemiology, Johns Hopkins Bloomberg School of Public Health, Baltimore, MD, USA;; ^4^ Department of Internal Medicine, Clinical Epidemiology and Biostatistics Unit, Makerere University, College of Health Sciences, Kampala, Uganda.

**Keywords:** sputum testing, Uganda, diagnostic yield, active case-finding, tuberculosis

Dear Editor,

Systematic screening or active case-finding (ACF) can reduce TB prevalence in high-burden settings,^1^ but the limitations of sputum-based diagnostic algorithms are a perceived barrier to widespread implementation.^2^ Current approaches typically identify high-risk individuals using non-sputum-based tests, such as symptom screening or chest X-ray (CXR), but subsequently require sputum-based molecular testing. The collection and provision of sputum samples is often difficult,^3,4^ and some large-scale screening efforts have successfully collected sputum from only a minority of eligible individuals.^1^ Most data on sputum production comes from populations of symptomatic people seeking health care, particularly people with HIV.^5,6^ Quantifying the extent to which sputum collection is a barrier to systematic screening and understanding its variation between risk groups, can inform planning for TB screening efforts in different populations and motivate the development of non-sputum-based TB diagnostics.^7^ Therefore, we examined the willingness and ability of individuals to produce expectorated sputum in a large community-based ACF effort in Uganda.

We conducted CXR-based TB screening in peri-urban Uganda as part of a pragmatic cluster-randomized trial of ACF (Clinicaltrials.gov: NCT05285202). Ongoing since June 2022, this analysis considers data through to February 29, 2024. All non-pregnant participants ≥15 years old, symptomatic or not, were offered CXR, with analysis by computer-aided detection (CAD:qXR, Qure.ai) in real-time. Participants with qXR scores above a specified threshold (initially set at 0.5, lowered to 0.1 by November 2022) were immediately requested to provide an expectorated sputum specimen for Xpert^®^ MTB/RIF Ultra (Cepheid, Sunnyvale, CA, USA) testing. Pregnant participants were asked for sputum without CXR. Participants were instructed to take two deep breaths, then cough, and provide at least 2 ml of expectorated sputum. Participants who struggled to expectorate were coached using an active cycle of breathing technique (ACBT).^8^ If they were still struggling, they were asked to try jogging and drinking water. No sputum induction was offered. Participants who could not provide sputum were referred to a local health facility for further evaluation. We estimated the proportion of participants with abnormal CXR who declined or failed to produce sputum. We categorized eligible participants by whether they attempted but were unable to expectorate (Group 1), declined to provide sputum (Group 2), or successfully provided expectorated sputum (Group 3). We compared characteristics of participants with/without sputum, comparing groups 1 and 2 versus group 3 and group 1 only versus group 3. Categorical and continuous variables were compared using χ^2^ tests and *t*-tests, respectively. We calculated exact binomial confidence intervals (CIs) and defined statistical significance as two-sided *P* < 0.05. This study was approved by the Institutional Review Boards at the Makerere University School of Public Health, Kampala, Uganda; and the Johns Hopkins School of Medicine, Baltimore, MD, USA. All participants provided informed consent.

Of 51,254 eligible consenting participants, 49,966 were non-pregnant, and 49,452 underwent CXR with valid results. Of those with CXR results, 7,510 (15.2%) had an abnormal CXR and were asked to provide sputum, of whom 6,746 (89.8%, 95% CI 89.1–90.5) successfully provided sputum; 213 (2.8%) declined to provide a sample; and 551 (7.3%) were willing but unable to expectorate. All but 18 (>99% of 6,746) sputum samples yielded valid Xpert Ultra results. Symptomatic individuals were more likely to provide sputum (91.8% of individuals with cough versus 88.3% with no cough, *P* < 0.01), as were men (91.5% of men vs. 88.0% of women, *P* < 0.01) and those with higher CXR scores (92.8% of those with qXR score ≥0.5 vs. 88.2% between 0.1 and 0.49, *P* < 0.01) (Table). Among 1,288 pregnant participants, 830 (64.4%, 95% CI 61.8–67.1) provided expectorated sputum, 76 (5.9%) declined, and 382 (29.7%) were unable to expectorate, and cough was associated with the provision of sputum (80.3% vs. 55.7%, *P* < 0.01).

**Table.** Characteristics of non-pregnant participants with abnormal chest X-ray results in eight communities in Uganda and their ability and willingness to provide expectorated sputum.

Characteristic^*^	Group 1: unable to expectorate	Group 2: declined to provide sputum	Group 3: provided expectorated sputum	*P-value* (Groups 1+2 vs 3)^†^	*P-value* (Group 1 vs 3)^†^
	*n* (%)	*n* (%)	*n* (%)	*n* (%)	*n* (%)
Total	551 (7.4)	213 (2.8)	6,746 (89.8)		
Female sex (*n* = 3,540)	312 (8.8)	114 (3.2)	3,114 (88.0)	<0.01	<0.01
Age, years, median [IQR] (*n* = 7,510)	50 [35–65]	50 [38–62]	51 [38–65]	0.08	0.17
Prior TB (*n* = 432)	20 (4.6)	13 (3.0)	399 (92.4)	0.07	0.03
Known TB contact within one year (*n* = 93)	10 (10.8)	2 (2.2)	81 (87.0)	0.38	0.21
Cough^‡^ (*n* = 3,310)	190 (5.7)	83 (2.5)	3,037 (91.8)	<0.01	<0.01
Fever (subjective)^‡^ (*n* = 1,750)	126 (7.2)	60 (3.4)	1,564 (89.4)	0.47	0.87
Night sweats^‡^ (*n* = 471)	39 (8.3)	12 (2.6)	420 (89.2)	0.63	0.43
Weight loss^‡^ (*n* = 309)	27 (8.7)	6 (1.9)	276 (89.3)	0.76	0.36
Known HIV infection^§^ (*n* = 645)	46 (7.1)	24 (3.7)	575 (89.1)	0.55	0.89
Current smoker^¶^ (*n/N* = 95/913)	1 (0.1)	–	94 (98.9)	0.43	0.61
qXR score ≥ 0.9 (*n* = 1,104)	43 (3.9)	24 (2.2)	1,037 (93.9)	<0.01	<0.01
qXR score ≥ 0.5 (*n* = 2,682)	138 (5.1)	56 (2.1)	2,488 (92.8)	<0.01	<0.01

* For binary variables (e.g., sex), numbers and percentages for one of the two categories (e.g., female) are presented, along with *P*-values comparing the distribution of the variable across the groups listed on the top row.

‡ Within the last 30 days.

† From χ^2^ test for categorical variables and *t*-test for a continuous variable.

§ By self-report.

¶ Only asked for participants enrolled after November 11, 2023.

IQR = interquartile range.

Our findings show that sputum can be successfully obtained from approximately 90% of adults found to have abnormal chest imaging during community-based TB screening and that high rates are achievable regardless of age, symptom or HIV status. Differences by sex, symptoms, and degree of CXR abnormality were small and unlikely to be clinically meaningful. Our 90% estimate is higher than often assumed when discussing the role of non-sputum-based tests and is higher than reported in some studies that may have imposed strict sputum quality requirements^1^ or may have deemphasized sputum collection because TB testing was an ancillary activity.^9^ However, other studies that measured TB prevalence similarly reported success (>90%) in sputum collection.^10,11^ For example, the 2014 Uganda national TB prevalence survey (which additionally engaged community health workers to follow-up with participants who had not yet returned sputum samples) collected sputum from approximately 93% of eligible participants.^11^ We collected sputum from a significantly lower proportion (approximately two-thirds) of pregnant individuals, to whom sputum testing was offered universally, than from people with abnormal X-rays. This lower sputum collection might be attributable to a lower prevalence of pulmonary abnormalities, lower motivation to expectorate in the absence of known CXR abnormalities, or lower priority given to TB testing during pregnancy. However, as pregnant individuals are at substantially increased risk of worse TB outcomes,^12^ this population may benefit from non-sputum-based diagnostics.

Our study had limitations. People with symptoms or underlying health problems may have self-selected to participate. For certain participants, the quality of expectorated sputum may have been suboptimal; however, even lower-quality sputum specimens may have good diagnostic yield in molecular testing. A prior ACF study in Uganda demonstrated that sputum specimens graded as salivary were nearly as likely to be Xpert-positive as higher-quality specimens.^13^ Furthermore, recent advances with tongue swabs as the diagnostic specimen suggest that the oral cavity can harbor enough *M. tuberculosis* DNA for samples to be useful in molecular testing for TB.^14^ Another limitation was distinguishing between inability and unwillingness to provide sputum. Besides, the ability to expectorate may depend on both participant motivation and health worker effort, which will likely differ across settings. Finally, our results may not generalize to settings with different air quality (ours was largely rural but with high biomass use^15^) or underlying lung health.

In summary, during systematic screening for TB in Ugandan communities, we found that most individuals with CXR abnormalities suggestive of higher TB risk were able to provide sputum – including most asymptomatic individuals. As such, systematic training of staff to encourage and coach all eligible individuals to provide a specimen should be prioritized. Our study demonstrates the feasibility of community-based screening for TB using existing sputum-based diagnostic tests. This underscores the importance of dedicated efforts to collect sputum from all individuals eligible for TB testing.
